# Rectal sunflower seed bezoar in an 11-year-old: A case report

**DOI:** 10.1177/2050313X251364028

**Published:** 2025-08-25

**Authors:** Sergine Cindy Zeufack, Patricia De Castro, Amandine Godier-Furnemont, Jennifer Reid, Camila Cribb Fabersunne, Valerie Gribben

**Affiliations:** 1 School of Medicine, University of California, San Francisco, CA, USA; 2 Department of Pediatrics, University of California, San Francisco, CA, USA; 3 Department of Surgery, University of California, San Francisco, CA, USA; 4 Department of Pediatrics, Zuckerberg San Francisco General Hospital, CA, USA

**Keywords:** gastroenterology, surgery, pediatrics, proctoscopy, bezoar, sunflower seed

## Abstract

Sunflower seed bezoars are an indigestible mass created after consuming several unshelled sunflower seeds. We present the case of an 11-year-old who visited the emergency department with abdominal pain, constipation, and rectal bleeding. The rectal exam findings were consistent with a presumed stool ball. Symptoms persisted even after enema and polyethylene glycol administration. The patient was discharged, and after an attempted manual self-disimpaction at home, the patient’s mother noted sunflower seed shell remnants in the stool. Upon further questioning, the patient endorsed consuming 10 ounces of salt and pepper-flavored unshelled sunflower seeds ~1 week before her first physician visit. Upon return to the emergency department, the patient underwent cross-sectional imaging, confirming the diagnosis of sunflower seed bezoar. Manual and instrumental disimpaction were performed under general anesthesia with symptomatic improvement. Seed bezoars should be considered for acute constipation refractory to laxatives in pediatric patients, especially after recent sunflower seed consumption.

## Introduction

Bezoars are collections of indigestible material, such as plant fibers (phytobezoars), hair (trichobezoars), and undigested milk in infants (lactobezoars) that can become obstructed along the gastrointestinal tract.^1,2^ Phytobezoars represent 40% of the reported common bezoars, with seed bezoars as a subset.^3^ In a systematic review, watermelon and sunflower seeds are the two most common types of seed bezoars.^4^ From pooled analysis of pediatric seed bezoar cases by Manatakis et al., 84% occurred in the rectum.^4^ Best practice management for rectal seed bezoars typically involves manual disimpaction under general anesthesia, as conservative measures such as stool softeners and enemas are often insufficient for resolution.^4^ Rectal sunflower seed bezoars are often discussed in international contexts,^5,6^ but few reports exist for cases in the United States. We report a pediatric patient with a rectal sunflower seed bezoar with corresponding imaging and discussion of management in a seed bezoar at high risk for perforation.

## Case presentation

### Day 1

An 11-year-old female with no significant past medical or family history presented to the emergency department with abdominal pain, 1 week of constipation, 2 days of rectal pain, and 1 day of rectal bleeding. Before presenting, the patient had received two doses of docusate and senna and a glycerin suppository with no improvement. In the emergency department, the patient had stable vital signs (temperature of 36.6 °C, elevated blood pressure of 133/87, heart rate of 91, respiratory rate of 18) and denied fever, nausea, and vomiting. The physical exam was notable for abdominal distention, mild generalized abdominal tenderness, and rectal tenderness, with a stool ball palpated during the digital rectal exam (DRE). The abdomen was soft without guarding or rebound, with no signs of peritonitis. The differential diagnosis at that point included constipation, hemorrhoids, diverticulitis, and small bowel obstruction. The presentation was deemed most concerning for uncomplicated constipation. The patient was administered an enema, which resulted in one stool and slight symptomatic improvement, and was subsequently discharged.

### Day 2

The following day, the patient received two doses of polyethylene glycol (PEG) at home. Unknown to the parent, the patient attempted a manual self-digital disimpaction, which caused bleeding and distress. The patient’s mother examined the patient and noted sunflower seeds in the stool and rectal area. Upon further questioning, the patient reported consuming two 5-ounce bags of salt and pepper-flavored sunflower seeds, including the shells, ~10 days before. In the past, the patient had spit out the shells when consuming sunflower seeds, but expressed that this time she did not because of the shells’ flavor and the effort of chewing. The patient and her mother returned to the emergency department for re-examination.

**Figure 1.**
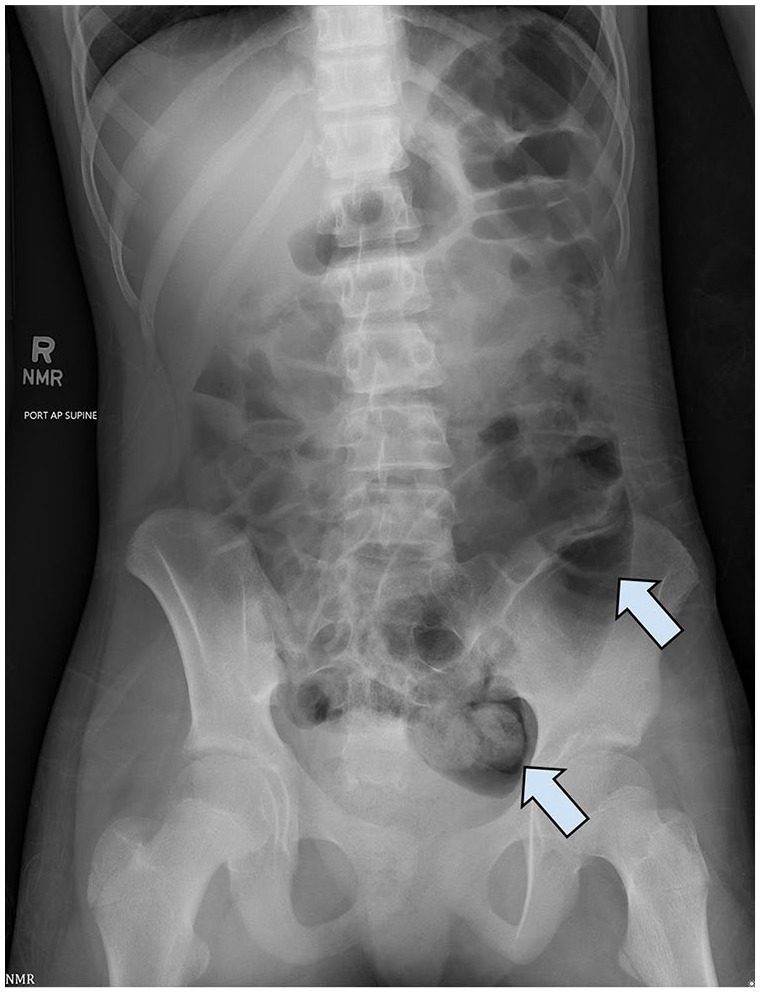
Abdominal X-ray showing dilated bowel loops (right arrow) with hyperechoic material in the distal left colon (left arrow).

The patient’s vitals on return showed normal temperature (36.6 °C), normal blood pressure (118/64), tachycardia (116 beats/min), and tachypnea (22 breaths/min). The physical exam was notable for a soft, distended, and diffusely tender abdomen with guarding but no rebound or peritonitis. DRE was deferred due to the patient’s severe discomfort, and the previous day’s DRE was referenced instead. External rectal exam showed rectal tenderness and no lesions or blood. Laboratory investigations revealed elevated white blood cell count (17,400 cells/μl) and hemoccult-positive stool. Abdominal X-ray showed diffusely dilated gas-filled bowels, most prominently in the distal left colon, where high-density material was noted (Figure 1). Multiple gas-fluid levels on cross-table lateral radiographs and absent rectal gas were concerning for obstruction.

**Figure 2.**
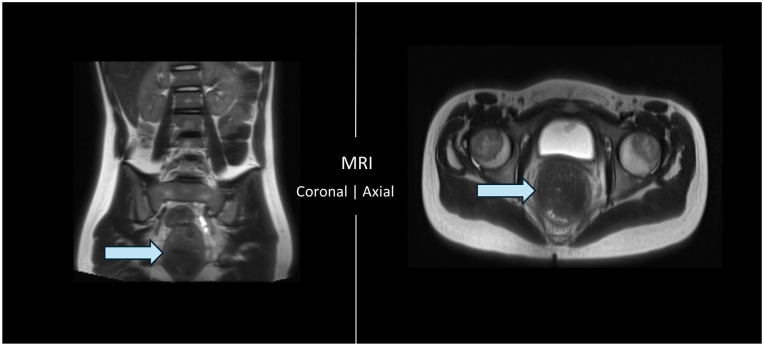
MRI of the abdomen without contrast from the coronal (L) and axial (R) perspectives showing dilated bowel loops and impacted rectum. Arrows point to fecal impaction in both perspectives. MRI: magnetic resonance imaging.

Though less likely for the patient’s age, an abdominal X-ray could not rule out volvulus. Magnetic resonance imaging (MRI) showed diffusely dilated gas-filled bowel loops with multiple air-fluid levels in the distal left colon and markedly impacted rectum (Figure 2). MRI confirmed the absence of small bowel distension or evidence of volvulus or perforation. The patient was admitted for definitive management.

History, imaging, and operating room findings were consistent with fecal impaction secondary to sunflower seed shell bezoar. Given the patient’s discomfort and the risk of perforation during disimpaction, the surgical team was consulted. The patient underwent manual disimpaction of the seed mass under general anesthesia in the operating room. The size of the bezoar was so large that it was difficult to extract the most proximal portion manually. To avoid rectal trauma and mitigate the risk of perforation, a rigid proctoscope was introduced so that the proximal portion could be removed under direct visualization using forceps. The operative findings included anal mucosa excoriation and a large mass of sunflower seeds in the rectum about 15 cm from the anal verge.

The treatment plan after surgical disimpaction included a mineral oil enema twice daily until soft bowel movements and topical lidocaine gel as needed. Hours after the procedure, the patient had a benign abdomen on physical exam and tolerated small meals. By the following day, the patient had a bowel movement and showed significant symptomatic improvement, no longer endorsing abdominal pain. The patient was discharged from the hospital and received counseling regarding the safe ingestion of sunflower seeds and a course of PEG to prevent new constipation while healing, to be continued until regular soft stools were achieved without persistent rectal pain. Two weeks after discharge, the patient had an in-person well-child appointment where she reported regular bowel movements and soft stools. Four months after the procedure, the patient reported completing the regimen and had no constipation or concerns.

## Discussion

Sunflower seed bezoars in the United States have been reported in pediatric patients as young as 35 months.^7^ A systematic review found the median age for children with gastrointestinal bezoars was 10 years.^4^ Pediatric seed bezoars occur most frequently in the rectum^4^; the pathophysiology is not fully understood but may be due to the collection of shell fragments in the rectal ampulla.^8^ Although the majority of seed bezoars are reported in other countries, sunflower seeds are widely consumed in the United States, so this diagnosis should remain on the differential. In general, bezoar risk factors can include prior gastric surgery, myotonic dystrophy, diabetes mellitus, hypothyroidism (congenital or Hashimoto’s thyroiditis), cystic fibrosis, poor mastication, and psychiatric illness.^1^ However, most patients with seed bezoars did not have risk factors.^4,9^ The most reported symptoms for seed bezoars in children were constipation (69%) and non-specific abdominal or rectal pain (19%).^4^ The patient’s symptoms and bezoar location were consistent with these articles. This report is the third reported pediatric case of rectal sunflower seed bezoar in the United States since 2000, raising concern for the risk of seed bezoars, especially those secondary to flavored sunflower seeds. The flavor of the shells played a role in the patient’s decision to consume a significant quantity. Similarly, a Canadian case report of flavored sunflower seed shell bezoar commented on flavor as a factor in making children and early adolescents more likely to ingest shells instead of spitting them out.^10^

Seed bezoars can be diagnosed by imaging, with abdominal X-ray and computed tomography (CT) as the most frequently mentioned imaging modalities.^4^ Though the patient’s initial presentation could have been consistent with uncomplicated constipation, given the patient’s persistent constipation even after at-home administration of a laxative, imaging was indicated to rule out more complicated etiologies of constipation. CT is used for improved bezoar localization and assessing the extent of obstruction and any acute bowel ischemia.^4^ MRI would be a reasonable alternative to CT for further analysis of X-ray findings without radiation exposure risk for a pediatric patient. Disadvantages of MRI include limited utility in acute bowel ischemia diagnosis^11^ and the potential need for sedation depending on patient age. Various studies in children^7,10^ mention key imaging findings for rectal seed bezoars, such as moderate to significant stool burden and large rectal masses. In Lyons et al.,^7^ an X-ray on initial presentation showed a nonobstructive gas pattern, while a subsequent X-ray after 2 days of stool softener showed mild gaseous distention in the large colon. In contrast, our report indicates bowel dilation and an obstructive bowel gas pattern as notable findings after 1 week of constipation.

Complications for seed bezoars include bowel perforation and, more rarely, colitis.^12,13^ Difficult disimpaction can cause tears in the anal mucosa.^6^ In this patient, it would not have been advisable to attempt bedside manual disimpaction given the significant pain, prolonged time course of symptoms, and previous failed attempts at disimpaction manually and with enemas. These unsuccessful efforts warranted surgical team involvement. Intraoperatively, there were many abrasions and excoriations seen in the anal canal, further highlighting the risk of perforation. Given the difficulty with manual disimpaction and the proximal extension of the bezoar, we used rigid proctoscopy to enable direct visualization and prevent further damage to the mucosa. Disimpaction under general anesthesia continues to be the recommended treatment method.^8^ As many reports show uncomplicated post-operative recovery, we recommend a phone check-in 1 month after discharge. It is reasonable to taper off or discontinue PEG at a follow-up visit if the patient is doing well symptomatically.

## Conclusion

A thorough history of symptoms, DRE, and imaging are required to successfully diagnose a potential seed bezoar and determine the extent of obstruction. In evaluating constipation in the pediatric population, the risk of sunflower seeds and other seed bezoars should be assessed by recent dietary history as well as possibly exacerbating bezoar risk factors, such as impaired gastrointestinal motility. Abdominal MRI can be considered as an alternative imaging modality for bezoar diagnosis in the pediatric population. Ultimately, after failed manual disimpaction, surgical consultation is essential for disimpaction under general anesthesia. Parents and guardians should be cautioned to supervise and guide the consumption of unshelled sunflower seeds, especially flavored ones.
